# Citrus disease detection using convolution neural network generated features and Softmax classifier on hyperspectral image data

**DOI:** 10.3389/fpls.2022.1043712

**Published:** 2022-12-07

**Authors:** Pappu Kumar Yadav, Thomas Burks, Quentin Frederick, Jianwei Qin, Moon Kim, Mark A. Ritenour

**Affiliations:** ^1^ Department of Agricultural and Biological Engineering, University of Florida, Gainesville, FL, United States; ^2^ USDA/ARS Environmental Microbial and Food Safety Laboratory, Beltsville Agricultural Research Center, Beltsville, MD, United States; ^3^ Department of Horticultural Sciences, University of Florida, Fort Pierce, FL, United States

**Keywords:** hyperspectral imaging, citrus canker, disease detection, food safety, convolution neural network (CNN), machine vision

## Abstract

Identification and segregation of citrus fruit with diseases and peel blemishes are required to preserve market value. Previously developed machine vision approaches could only distinguish cankerous from non-cankerous citrus, while this research focused on detecting eight different peel conditions on citrus fruit using hyperspectral (HSI) imagery and an AI-based classification algorithm. The objectives of this paper were: (i) selecting the five most discriminating bands among 92 using PCA, (ii) training and testing a custom convolution neural network (CNN) model for classification with the selected bands, and (iii) comparing the CNN’s performance using 5 PCA bands compared to five randomly selected bands. A hyperspectral imaging system from earlier work was used to acquire reflectance images in the spectral region from 450 to 930 nm (92 spectral bands). Ruby Red grapefruits with normal, cankerous, and 5 other common peel diseases including greasy spot, insect damage, melanose, scab, and wind scar were tested. A novel CNN based on the VGG-16 architecture was developed for feature extraction, and SoftMax for classification. The PCA-based bands were found to be 666.15, 697.54, 702.77, 849.24 and 917.25 nm, which resulted in an average accuracy, sensitivity, and specificity of 99.84%, 99.84% and 99.98% respectively. However, 10 trials of five randomly selected bands resulted in only a slightly lower performance, with accuracy, sensitivity, and specificity of 98.87%, 98.43% and 99.88%, respectively. These results demonstrate that an AI-based algorithm can successfully classify eight different peel conditions. The findings reported herein can be used as a precursor to develop a machine vision-based, real-time peel condition classification system for citrus processing.

## Introduction

Infections and other blemishes on the peels of citrus typically lower market value, and for certain locales, can prevent import or export. Citrus Canker and Citrus Black Spot (CBS) are among the latter category, but other lesser peel conditions such as melanose, greasy spot, wind damage and insect damage might only affect fruit price. Citrus Canker, included in this study, is a severe disease which has negatively impacted the Florida Citrus Industry for over two decades, it is usually characterized by conspicuous, erumpent lesions on leaves, stems, and fruit, and can cause defoliation, blemished fruit, premature fruit drop, twig dieback, and tree decline (Schubert et al., 2001). Considered one of the most devastating diseases that threaten fresh market citrus crops, canker at best reduces visual appeal to consumers, and at worst disqualifies entire shipments of fruit for export (Dewdney et al., 2022). Detection and removal of infected citrus at or before the packinghouse is essential to minimizing losses due to canker, but manual removal of infected fruit is inefficient and expensive. Automated identification of citrus peel conditions which not only detects more severe infections, but also differentiates between them and superficial blemishes would help assure fruit quality and safety, while also enhancing the competitiveness and profitability of the citrus industry.

Machine vision offers great potential for quality evaluation and safety inspection for food and agricultural products. Early work to inspect citrus for defects includes Sweet and Edward’s method to assess damages due to citrus blight disease on citrus plants using reflectance spectra of the entire tree (Sweet and Edward, 1986). Pydipati et al. (2006) utilized various textural features extracted with the color co-occurrence method (CCM) to identify diseased and normal citrus leaves using discriminant analysis. Blasco et al. (2007) showed that different spectra facilitate detection of different injuries and pathogens. Contemporary machine vision work with citrus canker inspection has harnessed Hyperspectral Imagery (HSI) and deep learning (DL) to improve and automate feature selection.

Instead of imaging with three broad frequency bands as typical cameras do, hyperspectral imaging cameras produce dozens of images in narrow bands, which can accentuate defects. HSI can also be used to evaluate the entire surface of food products and crops (assuming a rotating mechanism is present), as opposed to spot measurements with traditional visible/near-infrared spectroscopy. Food defects detected with HSI include bruises on cucumbers (Ariana et al., 2006), cracks in shell eggs (Lawrence et al., 2008), diseased poultry carcasses (Qin et al., 2008), and degradation of spinach leaves (Diezma et al., 2013). Contaminants detected with HSI include traces of nuts in wheat flour (Zhao et al., 2018), foreign objects in cut vegetables (Cho, 2021), contaminants on meat (Gorji et al., 2022) and fecal and ingesta contaminants on poultry carcasses (Park et al., 2007). Fresh fruits inspected with HSI include mandarins (Zhang et al., 2020), nectarines (Huang et al., 2021), jujubes (Pham and Liou, 2022), citrus (Gómez-Sanchis et al., 2008; Qin et al., 2013; Kim et al., 2014), tart cherries (Qin and Lu, 2005), pears (Lee et al., 2014), and mangoes (Rivera et al., 2014).

Out of the many DL algorithms, convolutional neural networks (CNNs) are widely used for object detection and image classification tasks (Guo et al., 2016; Liang et al., 2019; Krishnaswamy Rangarajan and Purushothaman, 2020). CNNs can outperform traditional machine vision methods, especially when processing time is a consideration (Fan et al., 2020). A few well-known CNN architectures researched for fruit inspection include ResNet (Mohinani et al., 2022), VGG-16 (Sustika et al., 2018) and AlexNet (Sustika et al., 2018; Behera et al., 2021; Ismail and Malik, 2022). With adequate training data, even several disease classes (26) and crop species (14) have been classified with 99.35% accuracy (Mohanty et al., 2016). The many applications of CNN for object detection and image classification include the disease classification in eggplant by Krishnaswamy Rangarajan and Purushothaman, 2020, classification of tobacco leaf pests by Swasono et al. (2019), kiwifruit defect detection by Yao et al. (2021), volunteer cotton plant detection by Yadav et al. (2022a); Yadav et al. (2022b); Yadav et al. (2022c); Yadav et al. (2022d), and identification of fecal contamination on meat carcasses by Gorji et al. (2022), etc. For citrus specifically, Syed-Ab-Rahman et al. (2022) distinguished CBS, canker, and citrus greening disease or Huanglongbing (HLB) on citrus leaves with a two-stage CNN, achieving mid-90’s detection accuracy on each of the three diseases. CNNs have been recently employed to detect, inspect, and track oranges on a rolling conveyor with 93.6% accuracy (Chen et al., 2021), to grade lemons with 100% accuracy (Jahanbakhshi et al., 2020), and to detect citrus fruit diseases (Dhiman et al., 2022). CNNs are often designed to balance speed with classification performance, as Fan et al. (2020) did when creating a custom architecture for an online apple sorting system. CNNs have a unique ability for automatic feature extraction which makes classification and detection tasks easier and autonomous.

Since the ability to automatically extract features allows for recognition of spectral features (Zhao and Du, 2016) in necessarily large HSI datasets, CNNs also complement HSI. CNNs were paired with HSI to detect anthracnose in olives (Fazari et al., 2021) with 100% accuracy for images taken 5 days after inoculation. Liu et al. (2018) employed an autoencoder combined with a CNN to learn features, quickly (14 ms per sample) and accurately (91.1% accuracy) detecting cucumber defects. VGG16 is one such widely used CNN architecture that is structurally simple and by successive means of 3x3 convolution improves the network’s performance for better feature extraction (Yang et al., 2021). It has been successfully used for many past image classification tasks, such as eggplant disease classification at 99.4% accuracy (Krishnaswamy Rangarajan and Purushothaman, 2020).

Citrus inspection has also employed HSI. Abdulridha et al. (2019) classified cankerous trees with 100% accuracy by imaging a citrus grove with an HSI camera on a UAV. Kim et al. (2014) used spectral information divergence (SID) to segment CBS from other orange peel conditions in HSI with 97% accuracy. But accurate classification of diseased citrus has been achieved with a small number of bands: Qin et al. (2011) used four, Lorente et al. (2013) used three plus the normalized difference vegetation index (NDVI), Bulanon et al. (2013) used two, and Jie et al. (2021) used three. However, all but the last of these extracted features without the aid of DL. And, the focus of Kim et al. (2014) was CBS detection, not classification of the other peel conditions.

The sheer size of HSI presents a processing challenge, which can be circumvented by reducing the number of bands from which features are extracted, while still retaining important spectral features. Spectral matching algorithms is one family of methods to segment images (Qin et al., 2009) and reduce dimensionality. van der Meer (2006) showed that SID outperforms other classical spectral matching algorithms.

Principal Component Analysis (PCA) is another popular means of reducing dimensionality, widely employed to extract and select features (Khalid et al., 2014; Jollife and Cadima, 2016). Su et al. (2014) used PCA as a standard by which to evaluate performance of other methods of selecting hyperspectral bands for display. Rodarmel and Shan (2002) and Farrell and Mersereau (2005) reduced hyperspectral imagery to fewer principal components using PCA without negatively impacting performance. PCA can also be employed to rank bands by contribution to the principal components, instead of projecting to a lower dimensional space. Li et al. (2019) used PCA loadings to select bands from hyperspectral imagery to inspect apples for decay. Li et al. (2011) used PCA to first select six bands from HSI cubes of oranges, and afterwards to extract the third principal component image. Eight types of defects were distinguished from healthy fruit with 94% accuracy. The advantage of using PCA is that the bands corresponding to the principal components are uncorrelated and therefore information contained in each of the bands is maximum (Cocianu et al., 2009).

For commercial fruit inspection applications, there are financial and performance reasons for eventually choosing a multi-spectral imaging (MSI) system over an HSI one. However, it is necessary to start with the full 92 HSI bands, determine the most prominent bands for classification and then implement those bands in MSI. Reduction from the original HSI’s 92 bands to just a few principal component bands is undesirable since these would still require the entire range of 92 bands to generate the reduced set of PCA bands. Instead, if a few salient spectral bands can be chosen from the 92, bandpass filters can capture images at only those wavelengths. PCA’s ubiquity, simplicity, and proven value for feature reduction make it an appealing technique for band selection, especially since it can also be employed to rank bands by contribution to the principal components, instead of projecting to a lower dimensional space, as nonlinear techniques do. Previous efforts to use a few selected HSI bands for online citrus disease inspection largely relied on manual feature extraction methods, which do not necessarily generalize to new defect conditions or other fruit varieties. On the other hand, a custom CNN can automatically extract optimal features. Therefore, the combination of a reduced set of spectral bands with a CNN-based feature extractor holds great potential for fast and accurate multiclass defect inspection. Moreover, many previous approaches dealt with only a few classes, while this study seeks to distinguish between eight unique peel condition classes with comparable or better performance.

The overall goal of this study was to use a DL-based approach with CNN to classify eight different peel conditions of citrus with HSI images. For this, we developed a custom CNN network that could do automatic feature extraction and classification of eight different peel conditions. The specific objectives were to (i) develop a CNN model and train it to classify HSI images of citrus with eight different peel conditions using 5 randomly selected bands (RS-5), (ii) use PCA-based 5 most contributing bands (henceforth abbreviated as PCA-5) and then train the developed CNN for classification of HSI images of citrus, (iii) study the effect of choosing the number of PCA-based bands on the performance metrics (accuracy, sensitivity and specificity) of the CNN model and (iv) compare the results of using RS-5 bands with that of PCA-5 bands for training the CNN model and classifying the HSI images of citrus.

## Materials and methods

### Hyperspectral imaging system

**Figure 1:**
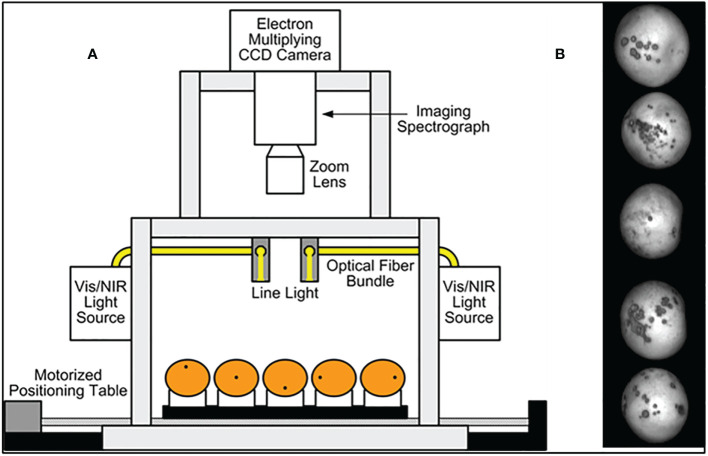
**(A)** Hyperspectral imaging system for acquiring reflectance images from citrus samples. **(B)** HSI single band sample image with canker peel condition on 5 fruit instances.

A hyperspectral imaging system was developed in previous work for acquiring reflectance images from citrus samples, and its schematic diagram is shown in **Figure 1** (Kim et al., 2001; Qin et al, 2008). This unit was based on the original concept by Kim et al. (2001). It is a push broom, line-scan based imaging system that utilizes an electron-multiplying charge-coupled-device (EMCCD) imaging device (iXon, Andor Technology Inc., South Windsor, CT, USA). The EMCCD has 1004×1002 pixels and is thermoelectrically cooled to -80°C *via* a double-stage Peltier device. An imaging spectrograph (ImSpector V10E, Spectral Imaging Ltd., Oulu, Finland) and a C-mount lens (Rainbow CCTV H6X8, International Space Optics, S.A., Irvine, CA, USA) are mounted to the EMCCD. The instantaneous field of view (IFOV) is limited to a thin line by the spectrograph aperture slit (30 μm), and the spectral resolution of the imaging spectrograph is 2.8 nm. Through the slit, light from the scanned IFOV line is dispersed by a prism-grating-prism device and projected onto the EMCCD. Therefore, for each line-scan, a two-dimensional (spatial and spectral) image is created with the spatial dimension along the horizontal axis and the spectral dimension along the vertical axis of the EMCCD.

The reflectance light source consists of two 21 V, 150 W halogen lamps powered with a DC voltage regulated power supply (TechniQuip, Danville, CA, USA). The light is transmitted through optical fiber bundles toward line light distributors. Two line lights are arranged to illuminate the IFOV. A programmable, motorized positioning table (BiSlide-MN10, Velmex Inc., Bloomfield, NY, USA) moves citrus samples (five for each run) transversely through the line of the IFOV. 1,740-line scans were performed for five fruit samples, and 400 pixels covering the scene of the fruit at each scan were saved, generating a 3-D hyperspectral image cube with the spatial dimension of 1740×400 for each band.

The hyperspectral imaging system parameterization and data-transfer interface software were developed using a SDK (Software Development Kit) provided by the camera manufacturer on a Microsoft (MS) Visual Basic (Version 6.0) platform in the MS Windows operating system (Kim et al., 2001). Spectral calibration of the system was performed using an Hg-Ne spectral calibration lamp (Oriel Instruments, Stratford, CT, USA). Because of inefficiencies of the system at certain wavelength regions (e.g., low light output in the visible region less than 450 nm, and low quantum efficiency of the EMCCD in the NIR region beyond 930 nm), only the wavelength range between 450 nm and 930 nm (totaling 92 bands with a spectral resolution of 5.2 nm) was used in this investigation.

### Citrus samples

Grapefruit is one of the citrus varieties that are most susceptible to canker which emerged as major Florida citrus disease in early 2000s. Ruby Red grapefruit, a high value citrus crop in Florida, was used in this study. Fruit samples with normal marketable, canker, five common diseased peel conditions (i.e., greasy spot, insect damage, melanose, scab, and wind scar) and with mixed peel conditions were collected at the University of Florida and first reported by Qin et al. (2008). The diseases on the fruit surface show different symptoms. Greasy spot, melanose, and scab are all caused by fungi, which generate surface blemishes that are formed by infection of immature fruit during the growing season. Greasy spot produces small necrotic specks, and the affected areas are colored in brown to black and exhibit greasy in appearance. Melanose is characterized by scattered raised pustules with dark brown to black in color. Scab appears as corky raised lesions usually with the color of light brown. Different from the fungal diseases, citrus canker is caused by bacteria, and it is featured with conspicuous dark lesions. Most circular in shape, canker lesions vary in size, and they are superficial (up to 1 mm deep) on the fruit peel (Timmer et al., 2000). Diameter of the canker lesions tested in this study was approximately in the range of 2-9 mm. Insect damage is characterized by irregular grayish tracks on the fruit surface, which are generated by larvae of leafminers that burrow under the epidermis of the fruit rind. Wind scar, which is caused by leaves, twigs, or thorns rubbing against the fruit, is a common physical injury on the fruit peel, and the scar tissue is generally gray.

**Figure 2:**
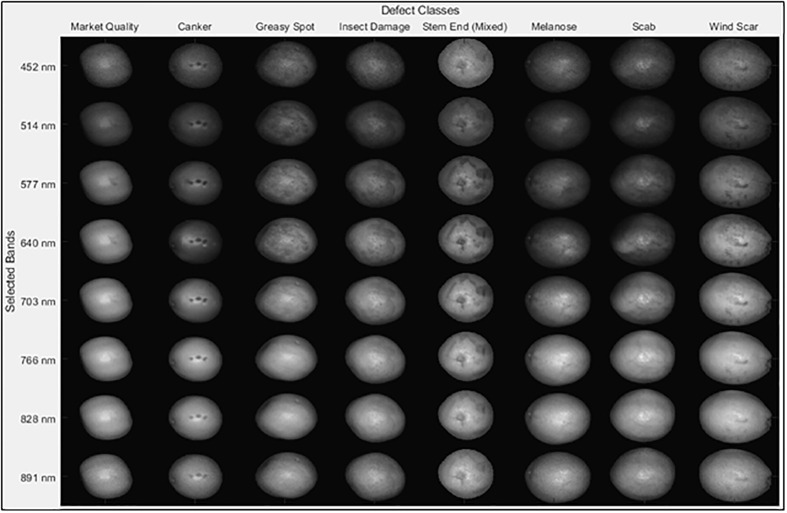
Plots of images of eight different peel conditions using the 23rd fruit from each class. Each HSI image contains five fruit samples for each of the peel conditions, but just one is displayed here.

Fruit samples were handpicked from a grapefruit grove in Fort Pierce, Florida during the spring 2008 harvest season. All the grapefruits were washed and treated with chlorine and sodium o-phenylphenate (SOPP) at the Indian River Research and Education Center of University of Florida in Fort Pierce, Florida. The samples were then stored in an environmental control chamber maintained at 4°C, and they were removed from cold storage about 2 hours before imaging to allow them to reach room temperature. During image acquisition, the citrus samples were placed on the rubber cups that were fixed on the positioning table (**Figure 1**) to make sure the diseased areas were on the top of each fruit as first reported in Qin et al. (2008). Each image band of the dataset is denoted by its center wavelength and is about 5.2 nm from adjacent bands. **Figure 2** displays one fruit from each of the eight defect classes of the dataset at a selected wavelengths representing the range of imaged wavelength bands. The MATLAB (The MathWorks Inc., Natick, MA) program was used to apply the same grayscale transformation to different images, to show varying levels of contrast among both fruits and defects. Being orange/yellow, the fruits themselves generally appeared brighter at longer wavelengths, but certain infections are more apparent in specific bands. Greasy spot and melanose are all but invisible at higher wavelength bands, but prominent at 577 nm. Scab and insect damage appear to contrast with healthy peel most at 640 nm. These suggest that classifying multiple peel conditions will require multiple wavelengths.

**Table 1** shows the number of HSI image samples that were collected for each of the eight different peel conditions, with each image including five fruits. This resulted in 1020 HSI image samples consisting of 5100 fruit specimens.

**Table 1:** Number of sampled HSI images with each peel condition.

Peel Condition	Label	Class Number	Images	Fruits/Image	Images with 5 Bands
Canker	CK	0	42	5	210
Greasy Spot	GS	1	24	5	120
Insect Damage	ID	2	12	5	60
Mixed Stem End	MD	3	12	5	60
Market Quality	MK	4	30	5	150
Melanose	MN	5	36	5	180
Scab	SB	6	36	5	180
Wind Scar	WS	7	12	5	60

### CNN with softmax classifier

**Figure 3:**
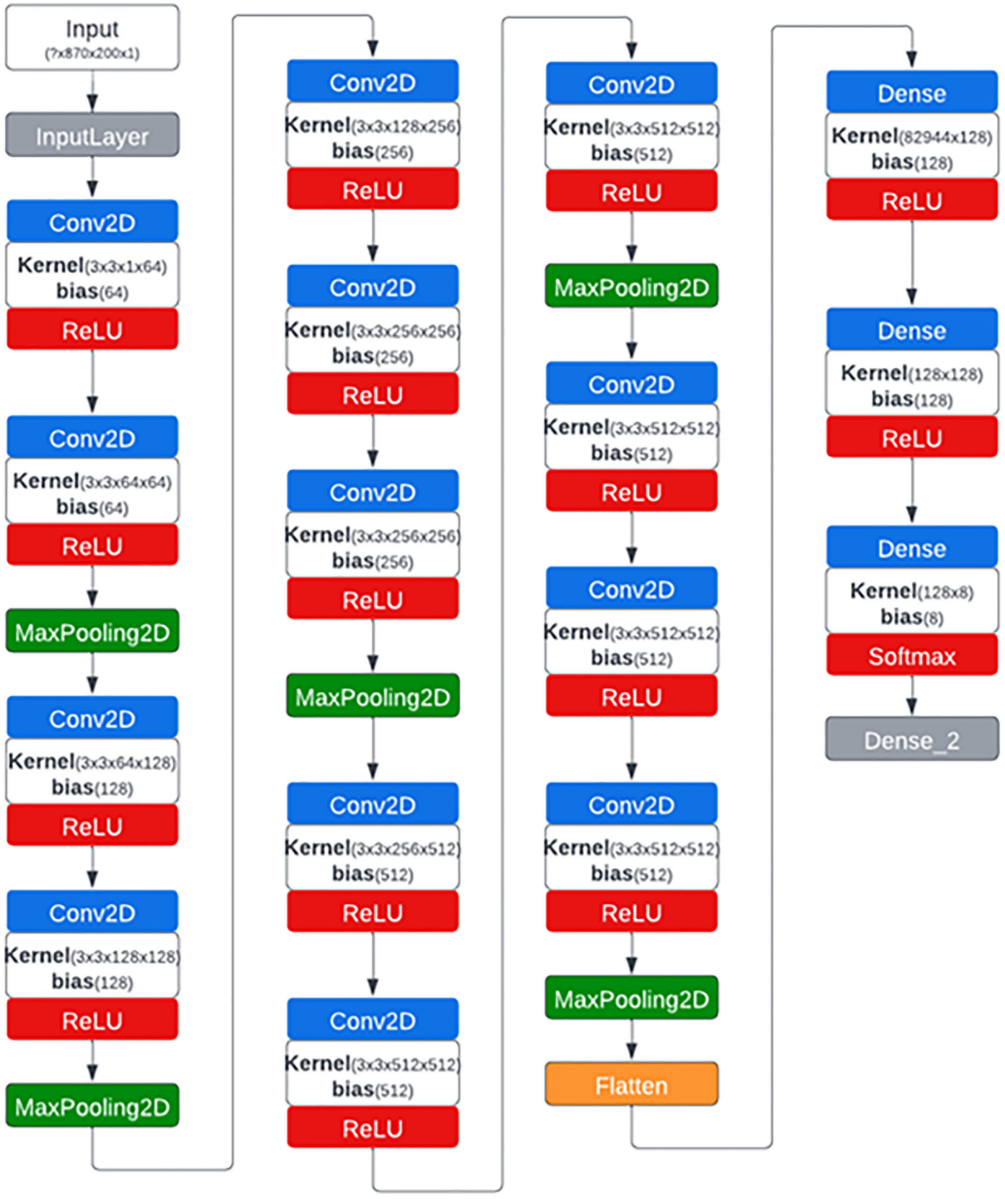
CNN network architecture generated by Netron visualization software that was used to classify hyperspectral images of citrus peels with eight different conditions.

The CNN architecture that we developed for this study is a modified version of VGG16 (Simonyan and Zisserman, 2014) that was developed by the Visual Geometry Group (VGG) in 2014 at Oxford University. The original architecture of VGG16 consists of 13 convolution layers, 3 fully connected (FC) layers and five max-pooling layers with softmax as the classifier. The original architecture was designed to accept input images of size 224x224 pixels which is why the trained weights are of similar size and therefore wasn’t suitable for our case applications for images of size 870x200 pixels. The customized network accepts images along a single channel which is why all the 92 spectral channels of each HSI input image were reshaped accordingly. To minimize the number of parameters significantly and hence the training and computation time, two of the three FC layers were reduced from 4096 to 128 units each and third to 8 units (corresponding to the number of classes) as more than 90% of the parameters and hence the weights are present in the last three FC layers (Braga-Neto, 2020). Padding of type “SAME” was used which ensured a convolution filter was applied to each of the input pixels. Adadelta (Zeiler, 2012) was used as an optimization algorithm for the training process with a learning rate of 0.01. The network architecture is shown in **Figure 3**.

The custom VGG16 network was attached to the softmax classifier that is a generalized version of logistic regression function used for multi-class classification (Stanford, 2013; Yadav et al., 2022a). Mathematically, a softmax classifier function is given as (Qi et al., 2018):


(1) $${\text{f}}_{\text{j}}\left(\text{z}\right)=\frac{{\text{e}}^{{\text{z}}_{\text{j}}}}{\sum_{\text{k}}{\text{e}}^{{\text{z}}_{\text{k}}}}$$

The softmax transforms the input into a probability distribution function that ranges from 0 to 1.

### Principal component analysis

Principal component analysis (PCA) is a multivariate statistical analysis technique that is used to analyze inter-correlated dependent variables in data. It is used to extract the most important information from data and then express them as a set of new orthogonal variables that are called principal components (Rodarmel and Shan, 2002). Assuming a set of bands as B_1_, B_2_, B_3…,_ B_p_ then the first principal component is the standardized linear combination of features Z_1_ = Ф_11_B_1_ + Ф_12_B_2_ +…. Ф_1p_B_p_ with the greatest variance. The components Ф_11_, Ф_12_, … Ф_1p_ are called loadings of the first principal component and Ф_1_= (Ф_11_+ Ф_12_+…. Ф_1p_) ^T^ is called the loading vector. PCA is a widely used technique in dimension reduction which eliminates highly correlated information from the ones with higher variance. Therefore, it is a commonly used tool in HSI based image processing to select the most important bands for classification purposes (Rodarmel and Shan, 2002). A scree plot in PCA analysis shows variance among each of the PC and therefore can be used to choose the number of components (Holland, 2019).

### Performance metrics

The CNN-based model was trained and validated on image datasets divided in the ratio 4:1 using the metrices accuracy, sensitivity and specificity as defined in equations (2)-(4). In the equations, TP, TN, FP, and FN represent True Positive, True Negative, False Positive and False Negative respectively.


(2) $$\text{Accuracy=}\frac{\text{TP+TN}}{\text{TP+TN+FP+FN}}$$

(3) $$\text{Sensitivity=}\frac{\text{TP}}{\text{TP+FN}}$$

(4) $$\text{Specificity=}\frac{\text{TN}}{\text{TN+FP}}$$

Sensitivity and Specificity are also known as true positive rate (TPR) and true negative rate (TNR) respectively. Besides these, we also used the area under receiver operating characteristic curve (AU-ROC) as the performance measure since it has been shown to give a better measure of classifier performance as compared to overall accuracy (Bradley, 1997). The ROC is plotted by putting TPR along y-axis and FPR (1-Sensitivity) along the x-axis and each point in the curve represents probability of classification at a particular threshold value. In other words, it considers the whole range of threshold values between 0 and 1 unlike the overall accuracy. The AU-ROC value represents degree of separability of a particular class. The advantage of using this metric is that it is not affected by class distribution, classification priori probability and misclassification cost (Xu-hui et al., 2009).

### Experiment setup

In this study we evaluated several band reductions approaches in conjunction with an CNN classifier to reduce computational demands. The first approach identified the PCA-5 bands and then used them to train the CNN model and classify the images using a CNN approach. This approach was later repeated using the 4,3,2 and single most contributing band to determine their classification accuracy in comparison to the PCA-5 bands. The entire process was repeated three times after which a fixed-effect test was done to analyze the effect of choosing the number of PCA-based bands on the accuracy, sensitivity, and specificity as explained by Borenstein et al. (2010). In a third approach, it was desirable to compare 10 sets of 5 randomly chosen bands (RS-5) with the PCA-5 results. In both cases, the CNN-based model was used to train and classify images. The resulting RS-5 accuracy, sensitivity and specificity were analyzed statistically and compared to the PCA-5 results. The first three approaches were based on groups of five fruit exhibiting the same peel conditions as shown in **Figure 1**. In the final approach we re-packaged the HSI dataset into individual fruits HSI images, so all training and classification were done on single fruit. Consequently, we have considered four test cases scenario shown in **Table 2**; (TC1) Determine the five strongest HSI bands among the 92 bands using PCA (referred to as PCA-5) and run CNN based training and classification to determine accuracy, sensitivity and specificity; (TC2) Compare results of TC1 with mean results from 10 RS-5 groups from the 92 original bands; (TC3) Compare CNN accuracy, sensitivity and specificity results from best five, four, three, two and single PCA selected bands (abbreviated as PCA-5, PCA-4, PCA-3, PCA-2, and PCA-1, respectively); and finally (TC4) Separate the original 5 fruit per HSI image data set into an individual fruit HSI data set, and compare performance of the PCA-5 CNN classifier for individual fruit to that of the five fruit classifier in TC1.

**Table 2:** Description of four types of experiments that were conducted in the study.

Experiment	Fruits Per Image	Description
TC1	Five	Train CNN with PCA-based 5 bands
TC2	Five	Train CNN with random 5 bands
TC3	Five	Train CNN with PCA-based 5,4,3,2,1 band
TC4	Single	Train CNN with PCA-based 5 bands

### CNN model training

The Google Colab Pro+ (Google LLC., 342 Melno Park, CA) platform was used to train the CNN model on an NVIDIA Tesla P100-PCIE GPU 343 (Santa Clara, CA) running Compute Unified Device Architecture (CUDA) version 11.2 and driver 344 version 460.32.03. The training was done with “EarlyStopping” enabled with a value of “patience” as 10 and “mode” set as “min”. This is used when the goal is to minimize the loss during the training process. By enabling this functionality, the training stops when the loss stops decreasing for any consecutive 10 iterations. Similarly, “ReduceLROnPlateu” functionality was used with a factor of 0.1 and patience of 7. This functionality reduces the learning rate by a factor of 0.1 for every 7 consecutive iterations without any improvement in the loss function. In each of the experiments, the CNN model was trained for 25 iterations with a batch size of 8 as the loss function converged in almost all the cases except in the case of TC4 in which the training iterations were increased to 35.

## Results and discussion

**Figure 4:**
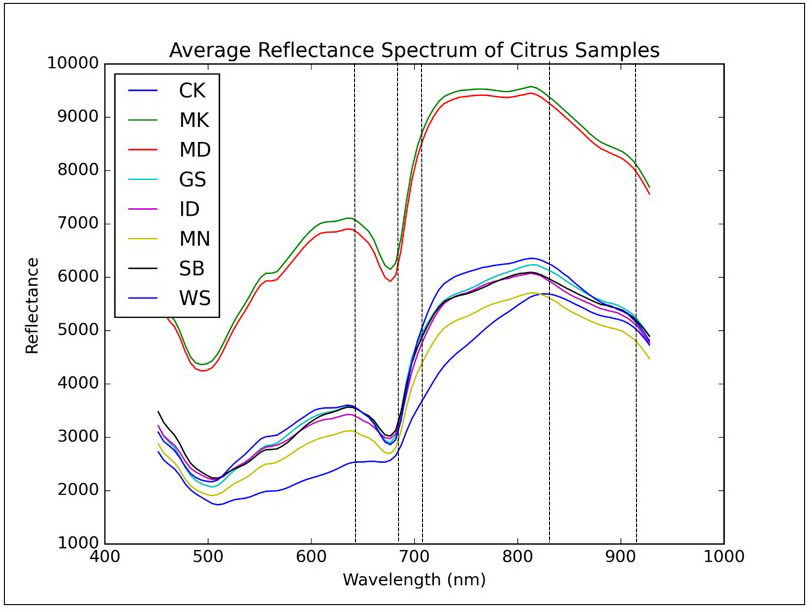
Mean reflectance spectra of grapefruits with eight different peel conditions. Dashed lines represent five wavelengths (666.15, 697.54, 702.77, 849.24 and 917.25 nm) selected by principal components analysis.

A typical reflectance spectrum of the grapefruit samples with normal and diseased conditions over the wavelength range between 450 nm and 930 nm are shown in **Figure 4** to demonstrate the general spectral patterns. Each spectrum plot in **Figure 4** was extracted from the hyperspectral image using an average spatial window covering 5×5 pixels for each peel condition. A cankerous sample was also included for the purpose of comparisons.

All the reflectance spectra were featured as two local minima around 500 nm and 675 nm due to the light absorption of carotenoid and chlorophyll A in the fruit peel, respectively. Chlorophyll is responsible for the green color of the citrus peel, while the yellow color of the fruit peel is related to carotenoid. The carotenoid and chlorophyll absorptions for greasy spot and canker were not as prominent as other peel conditions. Values of the reflectance for the diseased peel conditions were consistently lower than those from the normal fruit surface over the entire spectral region. Canker had the lowest reflectance except for the spectral region from 450 nm to 550 nm, in which greasy spot showed similar reflectance with canker. In the spectral region from 450 nm to 930 nm, the relative reflectance values of canker and normal peel were in the range of 12-43% and 31-66%, respectively, while other peel conditions generally had values in between.

For canker disease, once the bacterial pathogen invades the fruit peel, the region infected continually loses its moisture content through the season, and the lesions on the fruit surface usually become dark in appearance. This may be the reason for the low reflectance characteristics of the canker disease in visible and short-wavelength near-infrared region. The spectral differences between canker and normal as well as other diseased peel conditions provide a basis for detecting citrus canker using hyperspectral or multispectral imaging approach.

### TC1: CNN classification with PCA-5 bands

**Figure 5:**
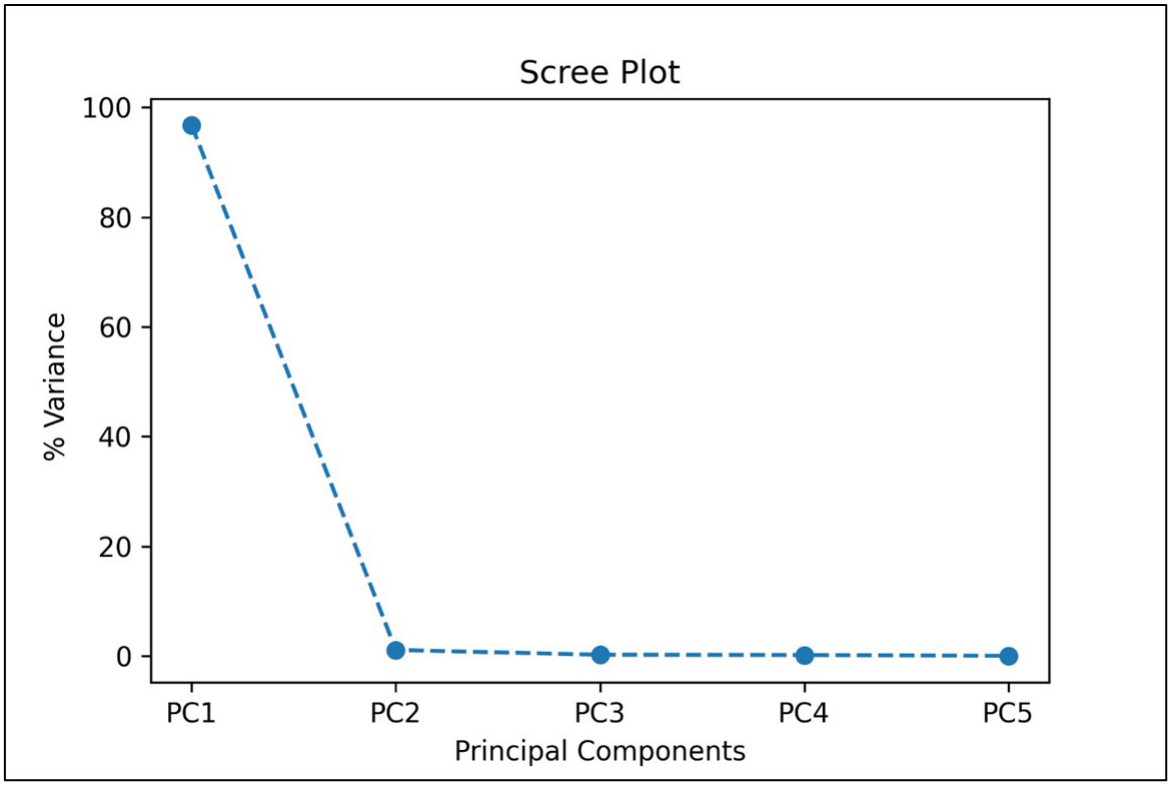
A scree plot of PCA analysis showing the percentage of variances accounted by each of the principal component.

In our first approach, TC1, we trained the CNN model using the five most important bands based on PCA, which would significantly reduce the computational complexity and thus training and validation time required when using all the 92 bands of the HSI images. We also considered five bands to be a reasonable transition from hyperspectral analysis (5 or more bands) to multispectral implementation (4 or less bands). From the given scree plot (**Figure 5**), it was found that the first 5 PCs accounted for a total of 98.32% of variance, with PC1 accounting for 96.74% by itself.

**Figure 6:**
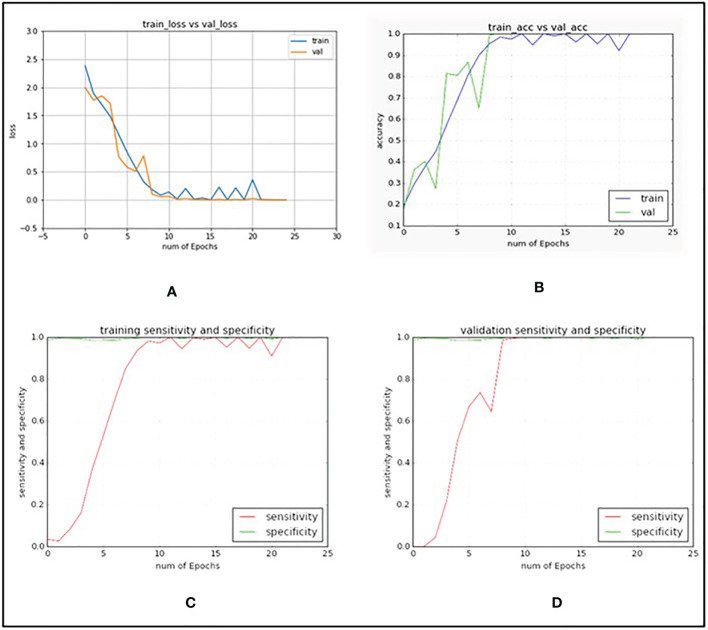
Graphs generated when custom VGG16 network was trained and validated with PCA-5 most contributing bands and with images containing 5 fruit instances (TC3). **(A)** CNN training and validation loss graph. **(B)** CNN training and validation accuracy graph. **(C)** CNN sensitivity and specificity graphs on training dataset. **(D)** CNN sensitivity and specificity graphs on validation dataset.

**Figure 7:**
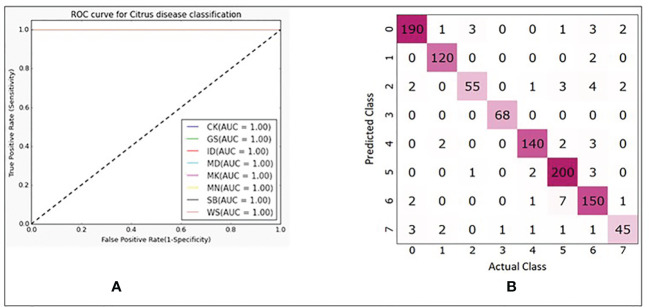
**(A)** ROC plots with corresponding AUC values for each class. **(B)** Confusion matrix showing number of correctly and misclassified images for each class (refer to **Table 1** for class numbers along x and y axis).

In **Table 3**, explained variance for each of the PC and corresponding bands are shown. From this, it was found that the most important bands were 42, 48, 49, 77 and 90. For PC2 and PC5 the same band 48 was the most important one, therefore, the second most important band for PC5 i.e., band 49 was chosen as the most important band in PC5. Once the PCA-5 bands were determined, the custom CNN model was trained to classify the images. Based upon three trials of training and validating with PCA-5 most important bands, the average accuracy, sensitivity, and specificity were found to be 99.84%, 99.84% and 99.98% respectively. The training and validation loss graphs that were generated on HSI dataset with 5 fruit instances are shown in **Figure 6A**, **B**. These graphs were generated during one of the three trails. The graphs converged within the 25^th^ iteration (epoch). The sensitivity and specificity were also tracked and plotted during the training process using both the training (**Figure 6C**) and validation datasets (**Figure 6D**). The AU-ROC as seen in **Figure 7A**, shows that the CNN model when trained with PCA-5 bands was able to discriminate all the classes almost equally on HSI images with 5 fruit instances over all the range of threshold values. The graphs also show that the ability of the model to detect TPR improves during the training process while its ability to detect TNR remains almost the same. The confusion matrix in **Figure 7B** shows the total number of correctly classified and misclassified images in the validation dataset. In this example trial run, all the 204 validation images were correctly classified.

**Table 3:** PCA Explained Variance for choosing the most important bands (PCA-5).

PC#	Bands	Wavelength (nm)	Explained Variance
1	**42**	**666.15**	**0.105053**
	43	671.38	0.105005
	41	660.92	0.105005
	40	655.69	0.104917
	39	650.46	0.104820
2	**48**	**697.54**	**0.281800**
	49 47	702.77 692.31	0.273946 0.272569
	50	708.00	0.253240
	51	713.23	0.225765
3	**77**	**849.24**	**0.184790**
	76	844.01	0.184519
	78	854.47	0.183582
	75	838.78	0.181392
	79	859.70	0.180943
4	**90**	**917.25**	**0.200681**
	91	922.48	0.200554
	89	912.02	0.198949
	88	906.79	0.195807
	87	901.55	0.193065
5	48	697.54	0.319693
	**49**	**702.77**	**0.265349**
	47	692.31	0.264272
	91	922.48	0.244818
	90	917.25	0.210242

Bold values refer to the most contributing wavelengths for each PC component.

### TC2: CNN classification with 10 replicates of RS-5

**Figure 8:**
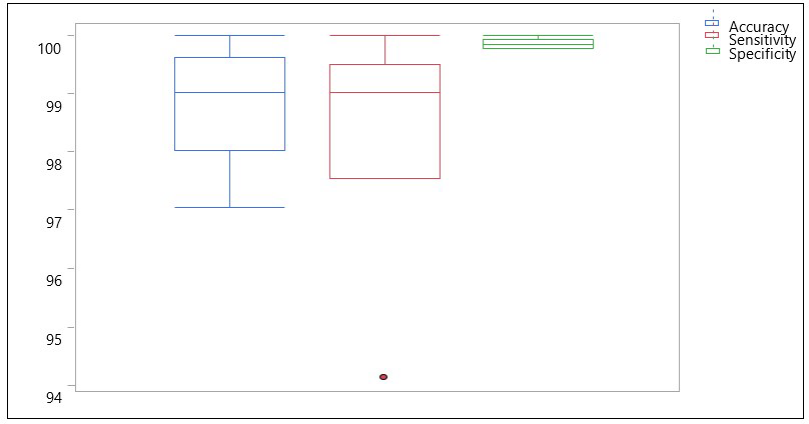
Box plots showing distribution of accuracy, sensitivity and specificity using 10 sets of RS-5 bands for the CNN model with images containing 5 fruits.

As a result of the high accuracy of TC1, the question was raised, would the CNN do just as well if 5 random bands were selected out of the 92. Consequently, the model was trained and tested for 10 repetitions (with 5 random combinations each time) and the results are shown in box plots (**Figure 8**). It was found that, the CNN was able to classify the HSI images at an average accuracy, sensitivity, and specificity of 98.87%, 98.43% and 99.88% respectively. This performance was better than our previous approach (spectral information divergence) which was used to classify canker versus non-canker peel conditions resulting in an overall accuracy of 96.2% (Qin et al., 2009). However, when compared to the PCA-5 bands (TC1), the performance was found to be slightly lower, indicating to use the TC1 approach.

Similarly, based on the AU-ROC, images belonging to the class MD were the most separable with a perfect AUC value of 1 followed by CK (AUC = 0.99818), GS (AUC = 0.98198), MK (AUC = 0.9712), MN (AUC = 0.94564), ID (AUC = 0.93866), WS (AUC = 0.93114) and SB (AUC = 0.88222). This was an interesting result as the number of images available for SB (180) were more than that of WS (60) and still the features of SB were less discriminative than that of WS. Similarly, even though ID, MD and WS had equal number of images available (60 each), the features of MD were more distinctive than those of ID and WS. This makes sense because MD always has a stem end feature along with other mixed peel conditions.

### TC3: Comparing results of CNN with PCA-5, PCA-4, PCA-3, PCA-2, PCA-1 bands

As a practical matter, it should always be considered how well the classifier would work if the imaging system was implemented in a multispectral application using four or less bands in a custom filtered multispectral camera. Consequently, in TC3, CNN performance was evaluated using PCA-5, PCA-4, PCA-3, PCA-2, PCA-1 band combinations (**Table 4**). These results were used to test the effect of number of PCA-based bands on the performance of CNN-based classifier using a fixed-effect model with JMP Pro version 16.1.0 software (SAS Institute, North Carolina, U.S.A.). Number of PCA-based bands had significant effects on all the three-performance metrics (accuracy, sensitivity, and specificity) of the CNN model in classifying HSI images of eight different peel conditions of citrus fruits as shown in **Table 5**, where the *p*-values for accuracy, sensitivity and specificity were found to be <.0001, <.0001 and <.0250 respectively at 95% confidence level (*α* = 0.05). However, number of PCA-based bands had relatively more significant effects on accuracy and sensitivity than the specificity because of higher variances among the group means for the formers than the latter as reflected by their corresponding F-ratios (**Table 5**). This essentially means that if one needs to obtain higher order of performance in classifying HSI images of citrus peels then one must be careful in choosing the number of PCA-based bands, since there is a significant drop off in performance when going from 4 bands to 3 bands. Thus, choosing the PCA-5 bands resulted in a mean accuracy, sensitivity, and specificity of 99.84%, 99.84% and 99.98% respectively, while 3 bands resulted in a mean accuracy, sensitivity, and specificity of 82.34%, 79.57% and 98.27% respectively, and single band resulted in a mean accuracy, sensitivity, and specificity of 45.53%, 35.61% and 96.21% respectively.

**Table 4:** Results of trained CNN with PCA-based bands.

Replicate	Number of Bands	Accuracy	Sensitivity	Specificity
1 2 3	5 5 5	100 99.51 100	100 99.51 100	100 99.93 100
1 2 3	4 4 4	99.39 98.17 97.56	99.39 96.95 96.95	99.91 99.83 99.83
1 2 3	3 3 3	72.36 88.62 86.18	65.32 87.1 86.29	97.47 98.85 98.5
1 2 3	2 2 2	81.71 81.71 80.49	73.81 73.81 76.19	98.47 98.81 98.29
1	1	36.58	34.09	90.91
2	1	43.9	29.55	98.7
3	1	56.1	43.18	99.03

**Table 5:** Fixed-effect model test to see if the number of PCA-based bands has any effect on accuracy, sensitivity, and specificity. .

Response variables	Source	Nparm	DF	Sum of Squares	F Ratio	p-value
Accuracy	Band_Num	1	1	4739.3928	45.3264	<.0001
Sensitivity	Band_Num	1	1	6896.5873	58.0544	<.0001
Specificity	Band_Num	1	1	23.549880	6.4177	<.0250

### TC4: comparing results of single fruit images with PCA-5 bands to that of TC1

**Figure 9:**
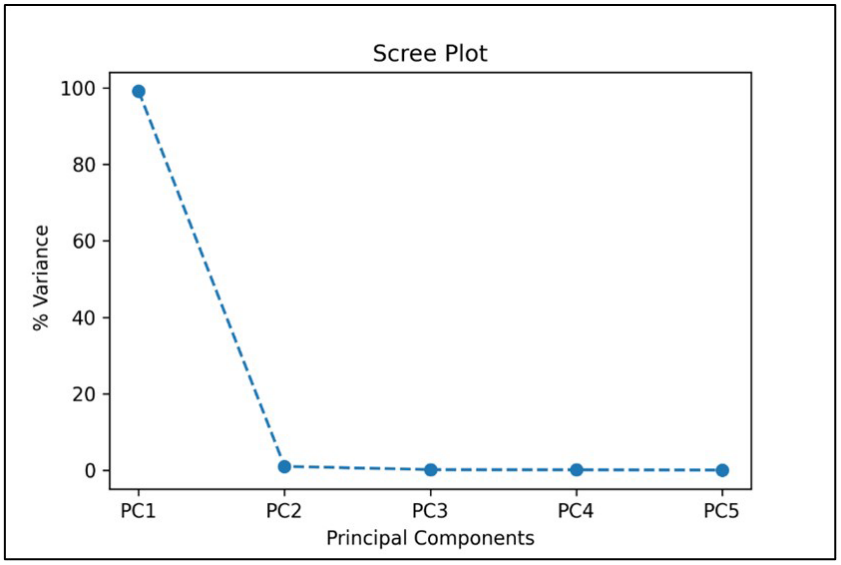
Scree plot showing the top 5 principal components when used with HSI images containing single fruit instances.

In the TC4 approach, we compared the results of CNN-based classifier by training and validating the model using HSI images containing single fruit instances using PCA-5 bands. The scree plot showing the 5 PCs with the corresponding variance percentages is shown in **Figure 9**. It was found that the top 5 PCs contributed to a total of 100% of the variances in the dataset and the 5 most important bands were found to be 42, 48, 69, 84 and 91 respectively. **Table 6** shows the values of explained variances corresponding to the top 5 bands based on the top 5 PCs.

**Table 6:** PCA Explained Variance for choosing the most important bands.

PC#	Band	Wavelength(nm)	Explained Variance
1	**42**	**666.15**	**0.104908**
	43	671.38	0.104885
	41	660.92	0.104863
	40	655.69	0.104788
	39	650.46	0.104712
2	**48**	**697.54**	**0.291559**
	49	702.77	0.283128
	47	692.31	0.279432
	50	708.00	0.259928
	51	713.23	0.229653
3	48	697.54	0.214328
	**69**	**807.39**	**0.20794**
	47	692.31	0.206631
	70	812.62	0.206057
	68	802.16	0.205691
4	**84**	**885.86**	**0.239097**
	85	891.09	0.236266
	83	880.63	0.235453
	86	896.32	0.232663
	87	901.55	0.226195
5	48	697.54	0.290974
	**91**	**922.48**	**0.277828**
	49	702.77	0.245112
	90	917.25	0.240679
	47	692.31	0.225747

Bold values refer to the most contributing wavelengths for each PC component.

**Figure 10:**
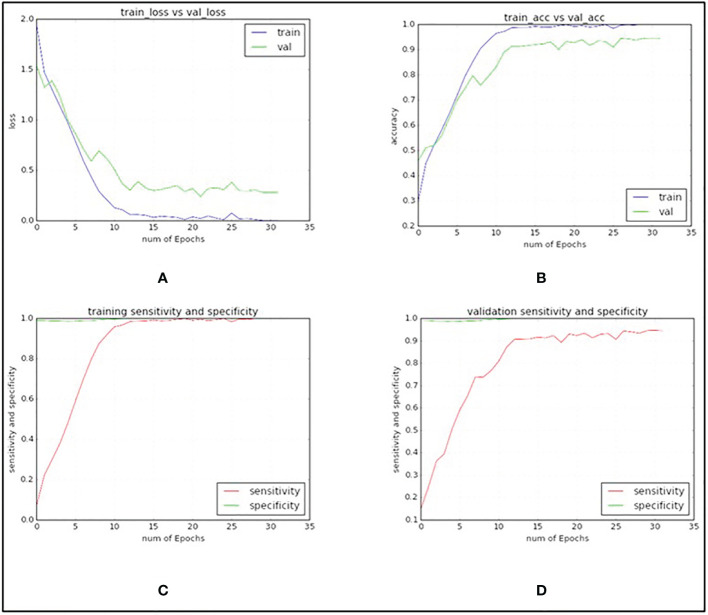
Graphs generated with PCA-5 bands on images with single fruit instances. **(A)** CNN training and validation loss graph. **(B)** CNN training and validation accuracy graph. **(C)** CNN sensitivity and specificity graphs on training dataset. **(D)** CNN sensitivity and specificity graphs on validation dataset.

The plots of using the PCA-5 bands (42, 48, 69, 84 and 91) for training and validating CNN model are shown in **Figures 10A–D**. The accuracy, sensitivity and specificity were found to be 98.53%, 98.53% and 99.80% respectively.

**Figure 11:**
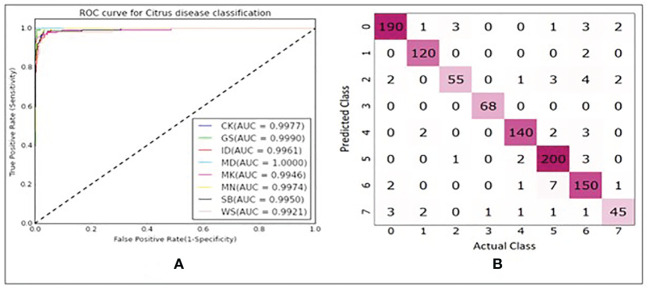
**(A)** ROC plots with corresponding AUC values for each class. **(B)** Confusion matrix showing number of correctly and misclassified images for each class(refer to **Table 1** for class numbers along x and y axis).

In **Figures 10A–D**, it is seen that the training iterations were slightly less than 35 as no improvement was observed in 10 consecutive iterations because “EarlyStopping” functionality was used with a patience value of 10. It is also worth noting that due to lack of convergence of the loss function within 25 iterations, the CNN model was trained for 50 iterations even though it was found to stop within the 35^th^ iteration (**Figures 10A–D**). In a trial of 3 repetitions, the average accuracy, sensitivity, and specificity were found to be 94.41%, 94.31% and 99.23% respectively. The loss functions on training data converged close to 0 while on the validation data it converged around 0.4 (**Figure 10A**). Based on the AU-ROC, images of MD class were the most accurately classified while the ones belonging to WS class were the least accurately classified (**Figure 11A**). The sensitivity on training dataset improved to almost perfect value of 1 (**Figure 10C**) while it converged around 90% on the validation dataset (**Figure 10D**). The specificity remained almost perfect throughout the training process as seen in the case of TC1. The diagonal elements in the confusion matrix show the number of correctly classified images and the non-diagonal elements represent the number of mis-classified images (**Figure 11B**). Even though ID class had the highest number of misclassified images (12), it still resulted in better performance than SB and WS over a range of thresholds as seen by AU-ROC (**Figure 11A**). When compared to the TC1, TC4 based approach resulted in a decrement of overall performance. In other words, the CNN resulted in more misclassifications of images with single fruit instances than the ones with 5 fruit instances. It is assumed that this may be because a greater number of features were able to propagate through the CNN network when trained with larger images (870x200 pixels) with multiple fruit instances in TC1 than in TC4 (174x200 pixels). It should be noted that classes MD, ID and WS had significantly fewer number of single fruit specimens than the other classes (MD, ID and WS had 60 images each while other classes had between 120 and 210). MD has very distinct features of nearly constant size due to stem end which likely attributed to its excellent accuracy, while ID and WS has highly variable feature size and shape due to nature of insect damage and wind scar. It is likely that the lower number of samples of ID and WS resulted in higher number of misclassifications of single fruit images in TC4 compared to the five fruit images in TC1. In addition, the SB class has strong similarity with CK and MN, which can be seen in **Figure 11B** confusion matrix where SB misclassified 7 times into MN and 2 times into CK. This could be improved possibly by a more balanced number of HSI for all classes. However, this remains to be a subject for future study.

## Conclusion

In this paper, we were able to show that the developed CNN models were able to classify HSI images of citrus peel with eight different conditions at a higher accuracy than our previously developed methods. We were also able to demonstrate the capability of PCA-based 5 most important band selection and then using them for training the CNN which resulted in a better performance than randomly selecting 5 bands. Apart from this, we were also able to demonstrate that using 5 fruit samples in each image results in slightly better classification accuracy (~ 4% higher) than the images with single fruit samples. Therefore, this study recommends using PCA for most important band selection, and then using those bands for training the CNN model to classify HSI images of citrus peels (possibly containing multiple fruit samples). WS, ID and SB had greater misclassification rates as they all appear spectrally similar. It was also observed that adjusting the threshold value of the classifier may slightly decrease the misclassification rate of ID. It, however, may not improve the performance of WS images as its misclassification rate remained the lowest in either of the cases.

The potential outcome of this study could be to deploy the trained CNN model on a machine vision based scouting systems in the field or at processing lines for real-time citrus peel condition detection at a faster speed and possibly better accuracy.

## Data availability statement

The raw data supporting the conclusions of this article will be made available by the authors, without undue reservation.

## Author contributions

First author: PY (methodology, software, validation, data curation, analysis, original draft preparation). Second author: TB (conceptualization, funding acquisition, methodology, investigation, review and editing). Third author: QF (data preparation, review and editing). Fourth author: JQ, MK and MR contributed equally (provided data, review and editing). All authors contributed to the article and approved the submitted version.
